# Immune Network Dysregulation Predicts Adverse Outcomes in Infective Endocarditis

**DOI:** 10.3390/tropicalmed11100265

**Published:** 2026-09-24

**Authors:** Isabela Galizzi Fae, Pedro Henrique Oliveira Murta Pinto, Gustavo Brandão Oliveira, Roni Arley Silva Duque, Fernanda Sophya Leite Cambraia, Lucas Chaves Diamante, Guilherme Lelis Costa, Andréa Teixeira-Carvalho, Deisy Morselli Gysi, José Luiz Padilha da Silva, Teresa Cristina Abreu Ferrari, Maria Carmo Pereira Nunes

**Affiliations:** 1 Programa de Pós-Graduação em Ciências Aplicadas à Saúde do Adulto, Faculdade de Medicina da Universidade Federal de Minas Gerais, Avenida Professor Alfredo Balena, 190, Santa Efigênia, Belo Horizonte 30130-100, MG, Brazil; isabelagalizzi@gmail.com (I.G.F.); phmurta@gmail.com (P.H.O.M.P.); brandaomed@gmail.com (G.B.O.); tferrari@medicina.ufmg.br (T.C.A.F.); 2 Hospital das Clínicas da Universidade Federal de Minas Gerais, Avenida Professor Alfredo Balena, 110, Santa Efigênia, Belo Horizonte 30130-100, MG, Brazil; roniduque@hotmail.com; 3 Departamento de Clínica Médica, Faculdade de Medicina da Universidade Federal de Minas Gerais, Avenida Professor Alfredo Balena, 190, Santa Efigênia, Belo Horizonte 30130-100, MG, Brazil; fernandacambraia.br@gmail.com (F.S.L.C.); lucasdiamante02@gmail.com (L.C.D.); guilherme_lelis_@hotmail.com (G.L.C.); 4 Diagnostic and Monitoring Biomarkers Laboratory of the Fundação Oswaldo Cruz, Instituto René-Rachou, Avenida Augusto de Lima, 1715-Barro Preto, Belo Horizonte 30190-002, MG, Brazil; atcteixeira@gmail.com; 5 Department of Statistics, Universidade Federal do Paraná, R. XV de Novembro, 1299, Centro, Curitiba 81530-015, PR, Brazil; deisy.gysi@ufpr.br (D.M.G.); jlpadilha@ufpr.br (J.L.P.d.S.)

**Keywords:** infective endocarditis, cytokines, immune networks, in-hospital mortality

## Abstract

Infective endocarditis (IE) is characterized by systemic inflammation, yet the contribution of cytokine network dysregulation to clinical outcomes remains poorly understood. We aimed to evaluate cytokine co-expression patterns in IE and identify immune network signatures associated with in-hospital mortality and major complications. We conducted a prospective cohort study of adult patients hospitalized with IE at a quaternary public hospital in Brazil (2012–2024). Serum cytokines and chemokines were measured at one to three time points during hospitalization. Weighted topological overlap (wTO) and Co-expression Differential Network Analysis (CoDiNA) were used to characterize cytokine interactions in baseline. Logistic mixed-effects models assessed associations between individual cytokines and in-hospital mortality. Of 305 screened patients, 160 met inclusion criteria (41 deaths; in-hospital mortality 25.6%). Survivors exhibited dense, integrated cytokine interaction networks, whereas non-survivors showed markedly fragmented networks with loss of connectivity across multiple cytokines. IFN-γ and IL-1RA formed a preserved but isolated axis in fatal cases, while IL-7 displayed outcome-specific connectivity in patients with infectious complications. A distinct triad involving IFN-γ, IL-1RA, and IL-6 differentiated patients with cardiac complications. In multivariable models adjusted for age, sex, and sampling time, CCL2 was independently associated with in-hospital mortality. Immune network disruption is strongly associated with adverse outcomes in IE. Distinct cytokine signatures, including altered interactions of IFN-γ, IL-1RA, IL-6, IL-7, and CCL2, identify high-risk patients and reveal potential biological pathways underlying clinical deterioration.

## 1. Introduction

Infective endocarditis (IE) remains a life-threatening condition associated with substantial morbidity and mortality despite advances in diagnostic imaging, antimicrobial therapy, and surgical management [1,2,3]. Over recent decades, the epidemiology of IE has shifted substantially, with increasing incidence among older adults, individuals with multiple comorbidities, and patients with prosthetic valves or cardiovascular implantable electronic devices [2,4,5]. *Staphylococcus aureus* has become a leading cause of community and healthcare-associated IE, contributing to its aggressive clinical course and rising complexity [2,6]. Although the burden of rheumatic heart disease-related IE has diminished in many regions, overall disease severity remains considerable [4].

The heterogeneous presentation of IE highlighted the need for accurate early risk stratification to guide clinical decision-making, including timely surgical intervention [4,7,8]. Current tools, including inflammatory biomarkers, echocardiographic findings, and clinical risk scores, offer valuable prognostic information but lack specificity and often fail to capture the dynamic immunopathological processes that drive complications and mortality [7,9,10,11,12]. Cytokines and chemokines play a central role in IE pathogenesis, mediating endothelial injury, vegetation formation, and systemic inflammation [13,14]. Prior studies have shown associations between elevated cytokine concentrations and poor outcomes, yet these investigations have largely relied on single time-point measurements obtained at diagnosis [15,16]. Because clinical deterioration or improvement in IE is strongly influenced by the patient’s evolving immunoinflammatory profile, longitudinal cytokine behavior may offer deeper insight into disease trajectory and therapeutic response, an aspect that remains largely unexplored.

Emerging systems immunology approaches indicate that immune dysregulation reflects a dynamic imbalance between pro- and anti-inflammatory pathways, in which the structure of cytokine interaction networks provides a more informative marker of immune failure and adverse outcomes than individual cytokine levels. Network-based analyses have demonstrated loss of immune coordination in severe infections such as sepsis and COVID-19, correlating with clinical deterioration and mortality [17,18]. Whether similar immune network disruptions contribute to adverse outcomes in IE remains unknown.

Therefore, this study aimed to characterize longitudinal profiles of 30 circulating cytokines, chemokines, and growth factors and to determine whether baseline cytokine co-expression network architecture was associated with in-hospital mortality and major complications in patients with infective endocarditis. By integrating quantitative biomarker profiling with co-expression network analysis, we sought to identify immune signatures that could improve risk stratification and enhance understanding of host-response mechanisms in IE.

## 2. Materials and Methods

This prospective cohort study enrolled consecutive adult patients admitted to a quaternary public hospital in Brazil between 2012 and 2024 with a diagnosis of IE, established according to the Modified Duke Criteria as updated in the 2023 European Society of Cardiology (ESC) Guidelines [2,19]. A control group of 49 healthy individuals was also included. The control group consisted of healthy blood donors and was included as a reference group for circulating cytokine, chemokine, and growth factor concentrations. A single blood sample was collected from each control to establish a baseline reference profile.

Eligible patients provided one to three blood samples. The first sample was obtained either at the time of diagnosis or within seven days of initiating antimicrobial therapy. For patients requiring early cardiac surgery, this initial sample was collected prior to the procedure. A second sample was collected 15 days after the first, and a third sample was obtained after completion of antimicrobial therapy and before hospital discharge. For individuals who died during hospitalization, only the samples collected prior to death were available for analysis.

Patients were excluded if the diagnosis of IE was ultimately rejected according to the Modified Duke Criteria, typically due to identification of an alternative infectious source [19]. Additional exclusion criteria included: (i) cardiac surgery performed before the first sample could be obtained and (ii) receipt of more than seven days of antibiotic therapy prior to enrollment.

A total of 305 adult patients were hospitalized with an initial diagnosis of IE. Of these, 22 were excluded after an alternative infectious source was identified. Among the patients with a final diagnosis of IE, 109 were not included due to inability to collect samples, most commonly because they had already received prolonged antimicrobial therapy or required urgent surgery and were in the immediate postoperative period. Fourteen additional patients were excluded due to insufficient clinical documentation (Figure 1). The final study cohort therefore comprised 160 patients with IE, including two patients who experienced recurrent episodes requiring readmission. Although samples were collected during both hospitalizations, each patient was counted once for clinical analyses.

Baseline demographic and clinical characteristics were obtained at hospital admission, and laboratory data were collected as part of routine care. All patients underwent transthoracic and/or transesophageal echocardiography when IE was clinically suspected. Blood samples were collected in serum tubes without anticoagulants at designated time points for the measurement of inflammatory biomarkers. All patients received guideline-directed therapy according to recommendations and were followed until hospital discharge for assessment of IE-related outcomes [2].

This study was approved by the Research Ethics Committee of the Federal University of Minas Gerais (UFMG) (protocol number: 78226524.1.0000.5149). All participants provided written informed consent prior to inclusion.

**Outcome definitions:** The primary outcome of the study was in-hospital mortality, defined as death from any cause occurring during the index hospitalization following the diagnosis of IE. The secondary outcome was the occurrence of cardiac or infectious complications attributable to IE, defined as follows:

**Cardiac complications:** Acute or progressive heart failure or cardiogenic shock typically resulting from severe valvular regurgitation due to infection-induced destruction of valve leaflets or supporting structures, as identified on transthoracic or transesophageal echocardiography, including prosthetic valve dysfunction presenting with severe regurgitation.

**Infectious complications:** Fever persisting for ≥10 days after exclusion of extracardiac causes; persistently positive blood cultures, defined as ≥2 positive cultures obtained >7 days apart despite appropriate antimicrobial therapy; septic shock; or perivalvular extension of infection, including abscess formation, fistula, or new-onset atrioventricular block.

### 2.1. Biomarker Measurement

The concentrations of chemokines, cytokines, and growth factors were measured and quantified using the Luminex assay (Bio-Rad, Hercules, CA, USA). This technique enabled the analysis of chemokines [CCL2, CCL5, CCL3, CCL4, CCL11, CXCL8 (IL-8), and CXCL10]; pro-inflammatory cytokines [interleukin (IL)-1β, IL-6, tumor necrosis factor-alpha (TNF-α), IL-12, interferon-gamma (IFN-γ), IL-17, and IL-15]; regulatory cytokines [IL-1 receptor antagonist (IL-1Ra), IL-4, IL-5, IL-9, IL-10, and IL-13]; and growth factors [basic fibroblast growth factor (FGF-basic), platelet-derived growth factor BB (PDGF-BB), vascular endothelial growth factor (VEGF), granulocyte colony-stimulating factor (G-CSF), granulocyte-macrophage colony-stimulating factor (GM-CSF), IL-7, and IL-2].

To determine the levels of these mediators, aliquots of 50 μL of serum were added to 50 μL of a mixture of capture beads conjugated with monoclonal antibodies specific to the cytokines, chemokines, or growth factors of interest and incubated for 90 min at room temperature, protected from light. Following incubation and washing, the beads were re-incubated with a cocktail of biotin-conjugated monoclonal antibodies specific to the human cytokines, chemokines, or growth factors and streptavidin-PE. After incubation, the beads were again washed with a buffer solution, centrifuged, and the supernatant carefully discarded. Subsequently, data acquisition and analyte levels were measured using the Luminex 200 system and Bioplex Manager software. The results were analyzed using Exponent 3.0 software and expressed in pg/mL.

### 2.2. Statistical Analysis

Descriptive statistics were summarized according to variable type and distribution. Categorical variables are presented as counts and percentages. Continuous variables are reported as mean ± standard deviation or median (interquartile range, IQR) depending on distribution. Group comparisons between patients who survived hospitalization and those who experienced in-hospital mortality were conducted using the χ^2^ test or Fisher’s exact test for categorical data. Continuous variables were compared using non-parametric methods.

To examine the longitudinal relationship between circulating cytokines and clinical outcomes, logistic or multinomial regression models, as appropriate, were fitted using repeated cytokine measurements obtained during hospitalization. Models were adjusted for age, sex, and sampling time point (first, second, or third sample) and incorporated random intercepts for patients and sampling occasions to account for within-subject correlation and repeated measurements. In addition to in-hospital mortality, longitudinal cytokine profiles were evaluated according to the occurrence of multiple complications, defined as the presence of at least two of the following three adverse events during hospitalization: embolic events occurring during treatment, infectious complications, and cardiac complications.

### 2.3. Network Analysis

To identify potential markers for poor prognosis, a co-occurrence network was built to assess the relationship between cytokine levels at baseline, collected within seven days of antibiotic initiation, and clinical outcomes. The co-occurrence networks were constructed using the weighted topological overlap (wTO), where the correlation between a set of cytokines was corrected using all the others present in the analysis, thus reducing noise and false positives and considering the commonalities of those interactions. For the network analysis, only the first blood sample collected from each patient was used. Thus, the weighted wTO and CoDiNA (Co-expression Differential Network Analysis) represent baseline cross-sectional cytokine co-expression networks and were not intended to assess longitudinal changes in network architecture [20]. CoDiNA compares two networks and identifies edges that are significantly similar, different, or specific to each network [21]. Cytokines are then classified into three categories based on the assessment of whether the observed edge distribution deviates from the expected distribution (*p* adj < 0.001). For the statistical analysis, SPSS software for Windows, version 23.0 (SPSS Inc., Chicago, IL, USA) and R software (version 4.1.1) were used, employing the following packages: wTO, CoDiNA, tidyverse, gtsummary, broom.mixed, lme4, lmerTest, igraph and glmmTMB.

## 3. Results

### 3.1. Baseline Patient Characteristics

A total of 372 samples were collected from patients with IE. Three samples were collected from 77 patients, two samples were collected from 54 patients, and one sample was collected from 33 patients. In the control group, one sample was collected from each of the 49 individuals.

Among the 160 IE patients included, 94 (58.7%) were male, with a median age of 58 years (IQR: 36–80 years). In the control group, 20 patients (40%) were men, with a median age of 41 years (IQR: 30.5–50.5).

The most frequent predisposing conditions among IE patients were cardiac implantable electronic devices (CIEDs) (40.8%), prosthetic valves (25.0%), and rheumatic heart disease (RHD) (20.0%). Common comorbidities included diabetes mellitus (21.9%) and chronic kidney disease (19.4%). Fever (75.6%), cardiac murmur (45.6%), and embolic events (30.0%) were the most common presenting features (Supplementary Table S1). *Staphylococcus aureus* was the most frequently identified pathogen (23.1%), followed by coagulase-negative staphylococci (19.4%) and aerobic Gram-negative bacteria (10.6%). Blood cultures were negative in 21.9% of cases. Echocardiography demonstrated valvular or perivalvular complications in 36.3% of patients.

During hospitalization, 41 patients (25.6%) died. Sepsis was the leading cause of in-hospital mortality (56.1%), followed by postoperative cardiac complications, stroke, and heart failure. Overall, 85% of patients experienced at least one complication, most commonly cardiac (45.6%), infectious (41.9%), or embolic (25.0%) events (Table 1). Valve surgery was performed in 36.9% of cases.

Clinical, laboratory, imaging characteristics, and complications stratified by in-hospital mortality are summarized in Table 1. Independent predictors of in-hospital mortality included older age, an unfulfilled surgical indication, and the occurrence of cardiac, infectious complications or embolic events during treatment.

### 3.2. Cytokine Profiles and Association with Mortality

Cytokine levels from the first blood sample were compared among IE survivors, non-survivors, and healthy controls (Table 2).

Cytokine levels were compared among three groups: patients with IE who survived hospitalization, IE patients who did not survive, and a control group of healthy individuals. Most biomarkers differed significantly among the three groups, as indicated by the *p*-values reported in Table 2. Among IE patients, significant differences were observed in the levels of CXCL8, CCL3, IL-6, IL-15, IL-5, G-CSF, and IL-2 between survivors and those who experienced in-hospital mortality.

### 3.3. Longitudinal Cytokine Profiles and Clinical Outcomes

Temporal trajectories of cytokine levels during hospitalization were evaluated according to in-hospital mortality (Figure 2). Models were adjusted for age, sex, and sampling time point (first, second, or third sample), and incorporated random effects for both patient and time. Outliers exceeding three standard deviations above the mean for each cytokine were removed to minimize the influence of extreme values.

Among all cytokines evaluated, CCL2 (MCP-1) demonstrated a significant association with in-hospital mortality. The odds of death increased by 33% for each 10 pg/mL increase in CCL2 concentration (OR = 1.33; 95% CI, 1.29–1.36; *p* < 0.001).

Longitudinal cytokine profiles were also evaluated according to the occurrence of multiple complications during hospitalization, defined as the presence of at least two of three adverse events: embolic events during treatment, infectious complications, and cardiac complications. Among the cytokines evaluated, IL-6 was significantly associated with the occurrence of multiple complications (OR = 3.86; 95% CI, 1.75–8.50; *p* = 0.021). The temporal trajectory of IL-6 according to the presence or absence of multiple complications is shown in Supplementary Figure S1.

### 3.4. Network Comparison

Network analyses were performed using the first blood sample collected from each patient. To identify potential markers for poor prognosis, a co-occurrence network was built using cytokine levels from the first blood sample collected from each patient. Thus, network analyses represented baseline cytokine co-expression patterns and did not incorporate serial measurements.

#### 3.4.1. In-Hospital Death

In survivors, cytokine interaction networks displayed a dense correlation structure, with IFN-γ and IL-1RA showing multiple connections to other biomarkers and a positive mutual association (weighted topological overlap [wTO] > 0). In contrast, the network among non-survivors was markedly sparser; IFN-γ and IL-1RA lost most interactions with other cytokines, although their mutual correlation was preserved. Integrated network analysis identified both conserved and outcome-specific interactions (Figure 3), indicating a loss of immune coordination in patients who died during hospitalization.

#### 3.4.2. Infectious Complications

Patients who developed infectious complications exhibited a substantially denser cytokine interaction network compared with those without complications, consistent with a more complex inflammatory response. IL-7 emerged as a key node uniquely associated with infectious complications, demonstrating multiple correlations in affected patients but minimal connectivity in those without complications. This divergent co-expression pattern suggests a potential role for IL-7 in dysregulated immune pathways underlying infectious progression in IE (Figure 4).

#### 3.4.3. Cardiac Complications

Distinct cytokine co-expression profiles were observed in patients with and without cardiac complications. In patients without cardiac complications, IFN-γ, IL-1RA, and IL-6 formed an extensive and interconnected network. In contrast, patients with cardiac complications showed an overall reduction in network density. However, CoDiNA analysis identified strong interactions among IFN-γ, IL-1RA, and IL-6 that were specific to patients with cardiac complications. This differentially co-expressed cytokine pattern effectively discriminated patients with and without cardiac complications, suggesting coordinated immune perturbations associated with cardiac involvement in IE (Figure 5).

## 4. Discussion

In this prospective cohort of patients with IE, we identified distinct inflammatory network signatures associated with in-hospital mortality and major IE-related complications. Using cytokine co-expression and CoDiNA differential network analyses, we demonstrated that survivors exhibited a more coordinated and interconnected cytokine response, whereas individuals who died or developed infectious or cardiac complications showed markedly disrupted network structures. Notably, IFN-γ and IL-1RA emerged as central cytokines whose connectivity patterns differed substantially between survivors and non-survivors, while IL-7 and the triad of IFN-γ, IL-1RA, and IL-6 displayed outcome-specific co-expression abnormalities in infectious and cardiac complications, respectively. Together, these findings suggest that the severity and progression of IE may be influenced not only by the magnitude of inflammatory activation but also by the integrity of cytokine interaction networks, providing novel insights that expand our understanding of the host response in IE.

Prior studies have emphasized the central role of inflammation in IE pathogenesis and prognosis [1,4,6]. Our findings extend this knowledge by demonstrating that adverse outcomes are associated not only with elevated cytokine concentrations but also with disruption of cytokine network architecture, suggesting that immune coordination, not merely immune activation, may influence clinical trajectories. Similar patterns of immune network disruption have been described in sepsis and other forms of severe infection [17,18], supporting the concept that immune dysregulation contributes to adverse outcomes.

A major observation of this study was the differential role of IFN-γ and IL-1RA in the mortality-associated networks. These cytokines, long recognized for their roles in macrophage activation and modulation of inflammatory injury [18,22], showed extensive interactions in survivors but markedly reduced connectivity in non-survivors. Similar immune network fragmentation has been described in severe infections such as sepsis and COVID-19 [17,18], supporting the hypothesis that immunological disarray contributes to adverse outcomes in IE.

Infectious complications were associated with an IL-7-centered adaptive immune dysregulation, whereas cardiac complications were characterized by a cytokine network analysis involving IL-6, IFN-γ, and IL-1RA, reflecting innate and adaptive immune activation and tissue injury [23,24,25]. IL-6 is consistently elevated in IE and is associated with acute valvular injury, heart failure, and poor prognosis, reflecting its role in promoting vascular inflammation, monocyte recruitment, and fibrosis [24,25,26]. IFN-γ and IL-1RA are also implicated in the modulation of inflammatory injury and macrophage activation, with higher levels of these cytokines linked to more severe disease and adverse outcomes [16,25]. The interaction of these cytokines in cardiac complications is supported by evidence that IL-6, IFN-γ, and IL-1RA are co-elevated in patients with severe or fatal endocarditis, and that IL-6 in particular is a robust marker of acute cardiac injury and adverse remodeling [9,25].

Interestingly, several immune mediators, including CXCL8, IL-1Ra, IFN-γ, IL-1β, and G-CSF, showed higher concentrations in controls than in patients with IE. These findings should be interpreted cautiously, as circulating concentrations of immune mediators are influenced by multiple factors beyond the presence of infection, including age and the timing of sampling. Moreover, a single circulating measurement may not fully reflect the complex and dynamic host immune response during bacteremic infection. Therefore, lower concentrations of individual mediators in IE patients compared with controls should not be interpreted in isolation as evidence of reduced immune activation.

The patterns observed in our network analysis align with established mechanisms of IE. Endothelial injury promotes bacterial adhesion, cytokine release, tissue factor expression, and vegetation growth composed of platelets, fibrin, and inflammatory cells [15,27]. Acute inflammation is mediated by TNF-α, IL-1, and IL-6, followed by activation of Th1 and Th2 adaptive pathways that facilitate pathogen clearance but can also drive systemic immunopathology when dysregulated [28].

Beyond network-based findings, longitudinal modeling revealed that CCL2 (MCP-1) was strongly associated with in-hospital mortality. CCL2 is a key chemokine that mediates monocyte recruitment to sites of endothelial activation and inflammation, driving the accumulation of monocytes and macrophages in infected cardiac tissue, which contributes to tissue injury and dysfunction [29].

Serial measurement and risk modeling in acute cardiovascular conditions, including infective endocarditis and acute coronary syndromes, have shown that CCL2 levels provide independent prognostic information beyond standard biomarkers such as CRP and BNP, and its persistent elevation identifies patients at higher risk for death and major adverse events [30]. This is supported by meta-analyses and cohort studies demonstrating that CCL2 is a robust predictor of cardiovascular mortality and organ dysfunction in infectious and inflammatory states [29]. Our findings reinforce the prognostic significance of CCL2 in IE and highlight its potential as an early biomarker for risk stratification.

The observed immune signatures parallel those seen in sepsis, where both hyperinflammatory and immunosuppressive phenotypes have been described [31]. Transcriptomic studies have identified shared pathogenic pathways between IE and sepsis, including upregulation of inflammatory mediators and immune exhaustion markers [32]. In our cohort, elevated IL-1RA and IL-9 in non-survivors further support the presence of immunosuppressive features in severe IE [33].

Taken together, these findings highlight the complex and dynamic nature of the immune response in IE. Traditional prognostic markers, including heart failure, persistent infection, and embolic events, remain fundamental but may not fully capture the immunological heterogeneity underlying disease progression [7]. The pathogenesis involves not only direct tissue injury and embolic phenomena but also maladaptive immune responses, such as hyperinflammation or immunosuppression, which can drive organ dysfunction and mortality even in the absence of overt heart failure or persistent bacteremia [34]. Furthermore, the variability in immune signatures across causative microorganisms and host backgrounds underscores the heterogeneity of the immunopathology of infective endocarditis (IE). This heterogeneity suggests that reliance solely on traditional clinical and laboratory markers may fail to identify patients at increased risk due to underlying immune dysregulation [34]. For instance, IE caused by *Serratia marcescens* may follow a fulminant course and have been associated with in-hospital mortality rates of up to 50% [35]. In contrast, IE caused by HACEK organisms is generally associated with a more favorable prognosis, with reported in-hospital mortality rates of approximately 2% [36]. This supports the need for integrated approaches that combine clinical, microbiological, and molecular data to more accurately assess prognosis and guide personalized therapy in IE.

Network-derived cytokine signatures may offer additional prognostic value and could support multidisciplinary Endocarditis Teams in deciding on early surgical intervention, intensive monitoring, or adjunctive therapies. Longitudinal cytokine assessment during hospitalization may also provide dynamic insights into treatment response.

This study has several strengths, including its prospective design, standardized application of updated Modified Duke Criteria, and comprehensive inflammatory biomarker profiling. The use of advanced network-based approaches provides insights not achievable with conventional biomarker analyses alone.

Nonetheless, several limitations should be acknowledged. This single-center study may limit generalizability, although the cohort was large and well characterized. Limited sample availability in patients requiring urgent surgery or with prolonged antibiotic exposure may have introduced selection bias. Network analyses identify associations but cannot establish causality. Although serial samples were collected, wTO and CoDiNA analyses were restricted to the first sample, providing a cross-sectional assessment of baseline cytokine network architecture and not capturing temporal changes during treatment. Additionally, differences in age and sex between patients and controls may have introduced residual confounding, as both factors influence immunosenescence and baseline cytokine profiles and were not accounted for in the between-group analyses.

## 5. Conclusions

This study demonstrates that adverse outcomes in IE, including mortality, infectious complications, and cardiac complications, are associated with disrupted cytokine co-expression networks and specific inflammatory signatures involving IFN-γ, IL-1RA, IL-6, IL-7, and CCL2. Network-based immunophenotyping may offer a promising avenue to improve risk stratification and guide future mechanistic and translational research in IE.

## Figures and Tables

**Figure 1:**
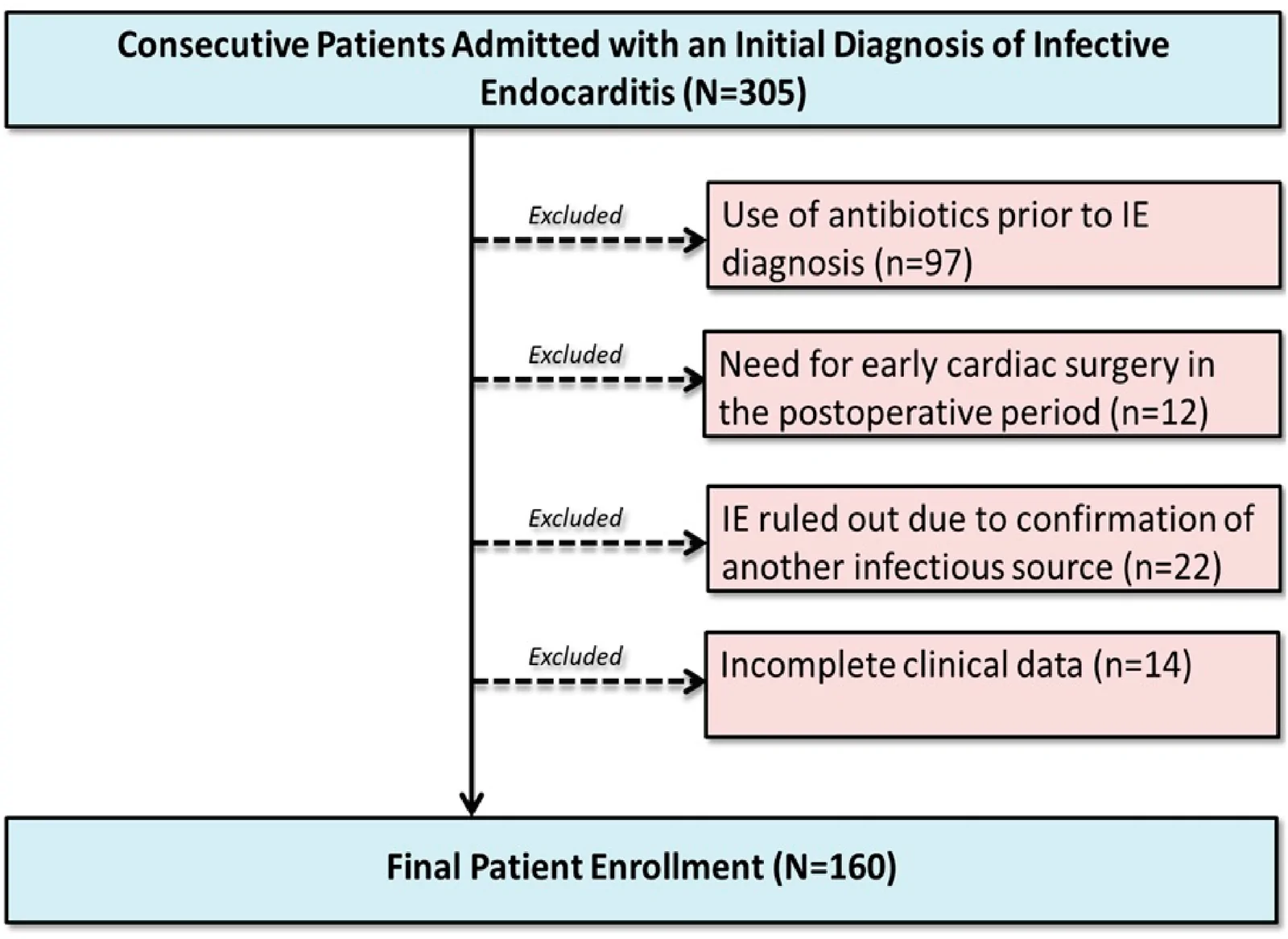
Flowchart of patient enrollment.

**Figure 2:**
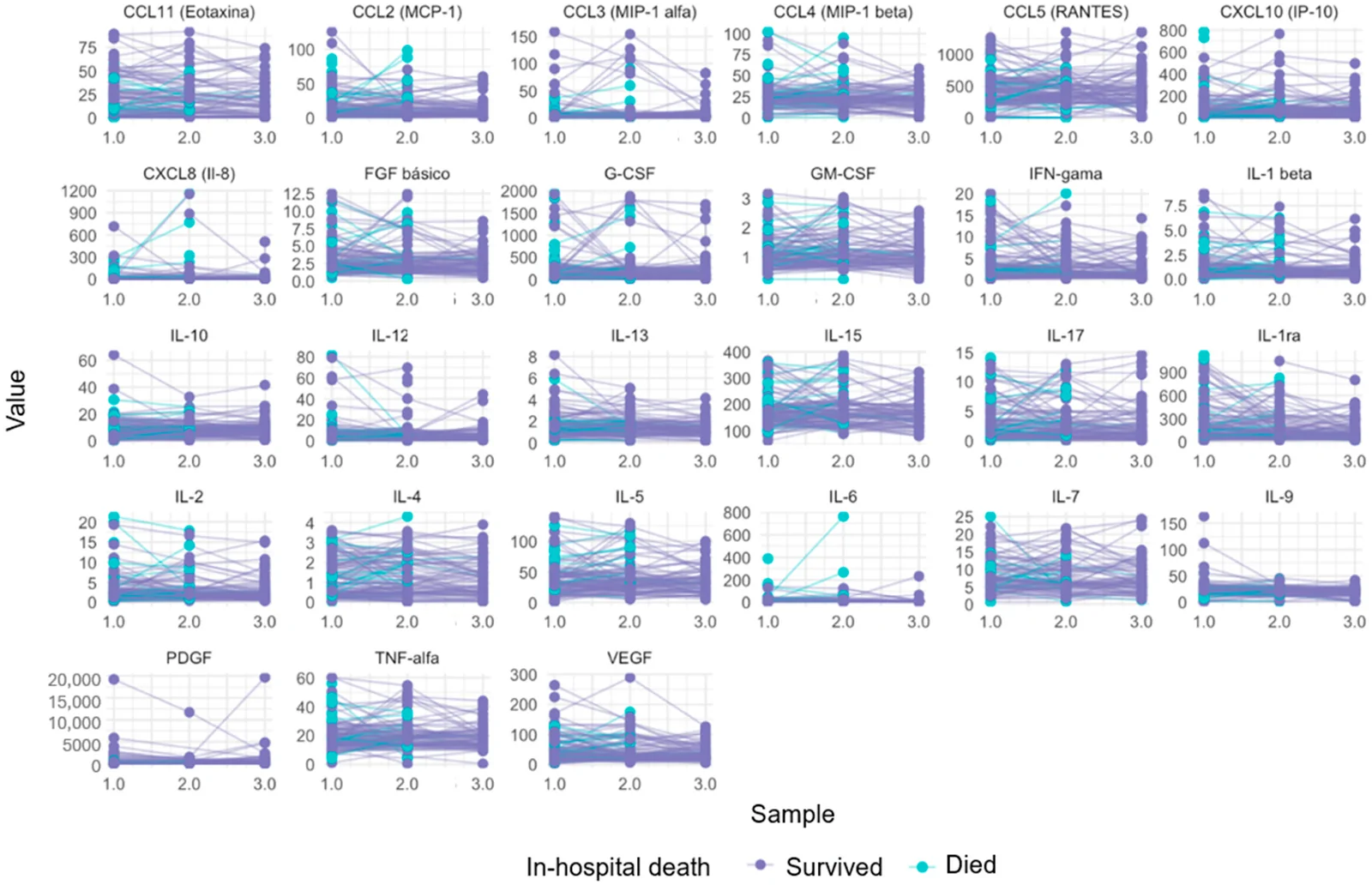
Temporal trajectories of cytokine levels among survivors and non-survivors in IE patients. Cytokine levels during hospitalization. The numbers on the *x*-axis correspond to each cytokine sampling time point (first, second, or third), while the *y*-axis represents cytokine levels. The purple color represents cytokine levels in patients who survived, and the green color represents cytokine levels in patients who died during hospitalization. Among cytokines, CCL2 (MCP-1) was associated with in-hospital mortality (OR = 1.33; 95% CI, 1.29–1.36; *p* < 0.001).

**Figure 3:**
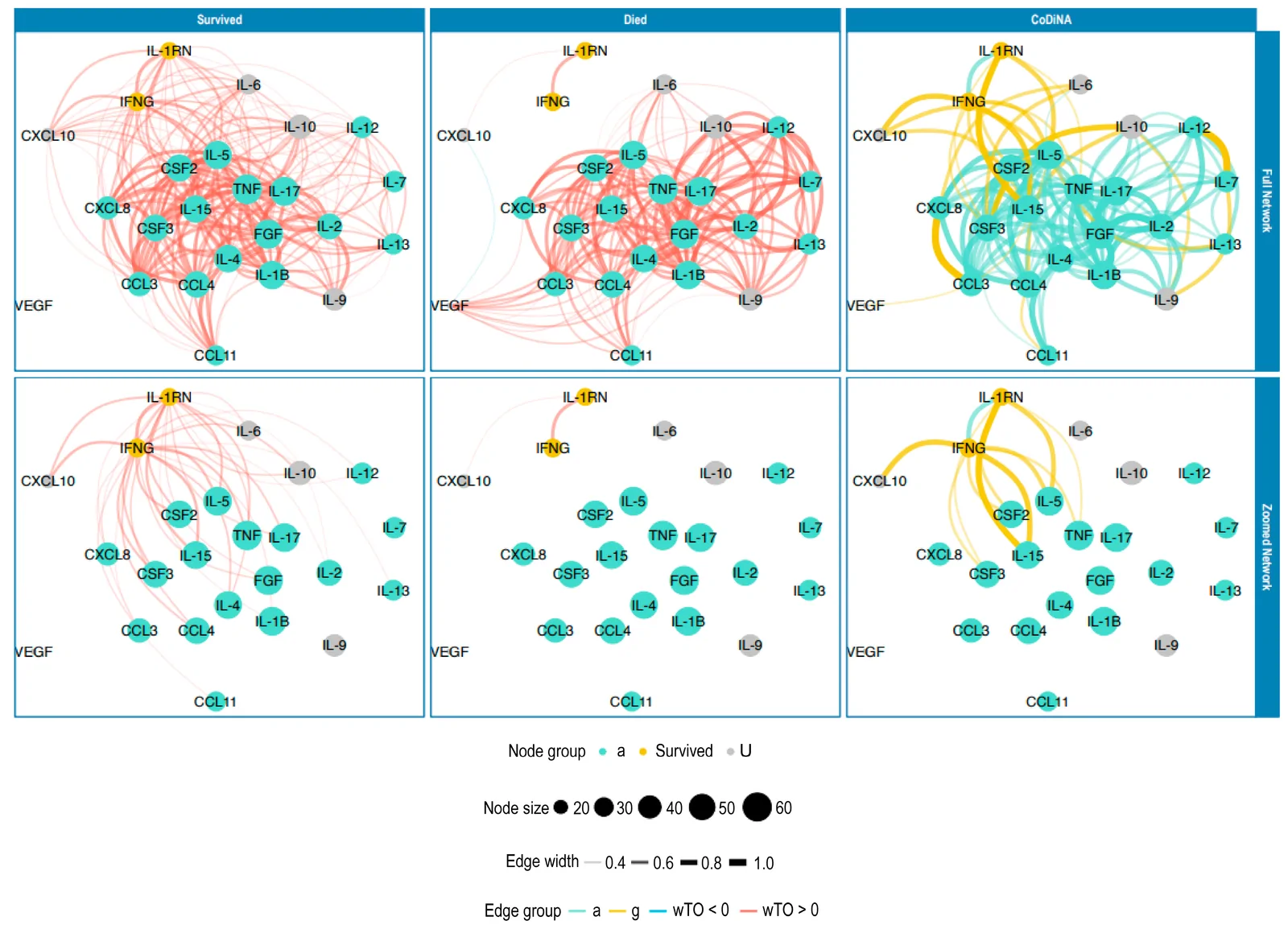
Cytokine network analysis according to survival status. Full (top) and zoomed (bottom) networks are shown for survivors, patients who died, and the differential CoDiNA network. Survivors ex-hibited a denser network with a greater number of cytokine interactions than patients who died. CoDiNA identified differential network patterns involving IFN-γ and IL-1RA. Node colours indi-cate CoDiNA node groups, node size reflects connectivity, and edge width represents interaction strength. Red and green edges represent positive and negative correlations, respectively, whereas yellow edges highlight differential interactions identified by CoDiNA.

**Figure 4:**
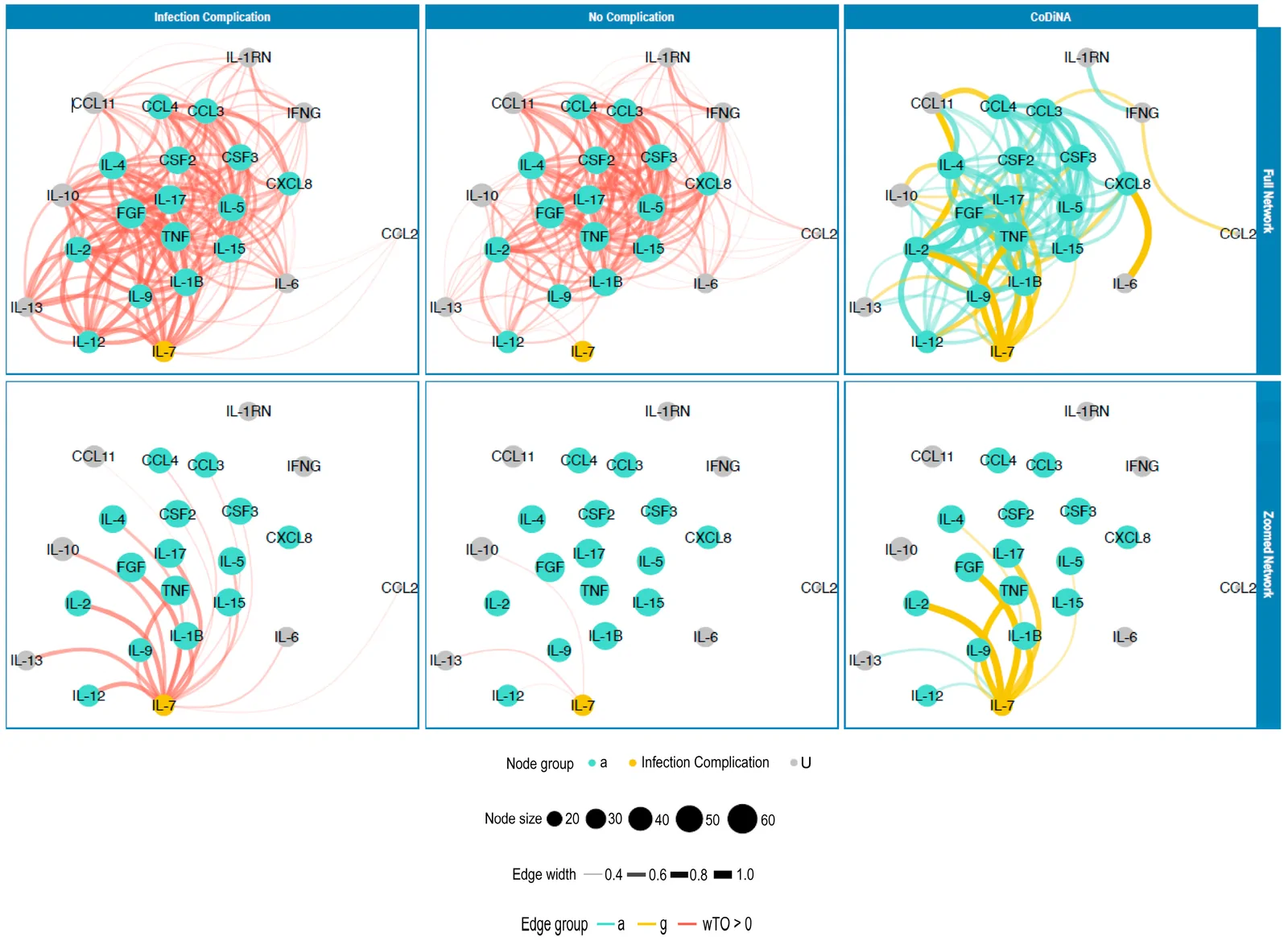
Cytokine network analysis according to infectious complications. Full (top) and zoomed (bottom) networks are shown for patients with infectious complications, those without complications, and the differential CoDiNA network. Patients with infectious complications exhibited a denser cyto-kine network, with a greater number of interactions than those without complications. CoDiNA identified differential interaction patterns, with IL-7 emerging as a prominent node in the network associated with infectious complications. Node colours indicate CoDiNA node groups, node size reflects connectivity, and edge width represents interaction strength. Red edges denote positive correlations, green edges represent negative correlations, and yellow edges represent differential interactions identified by CoDiNA.

**Figure 5:**
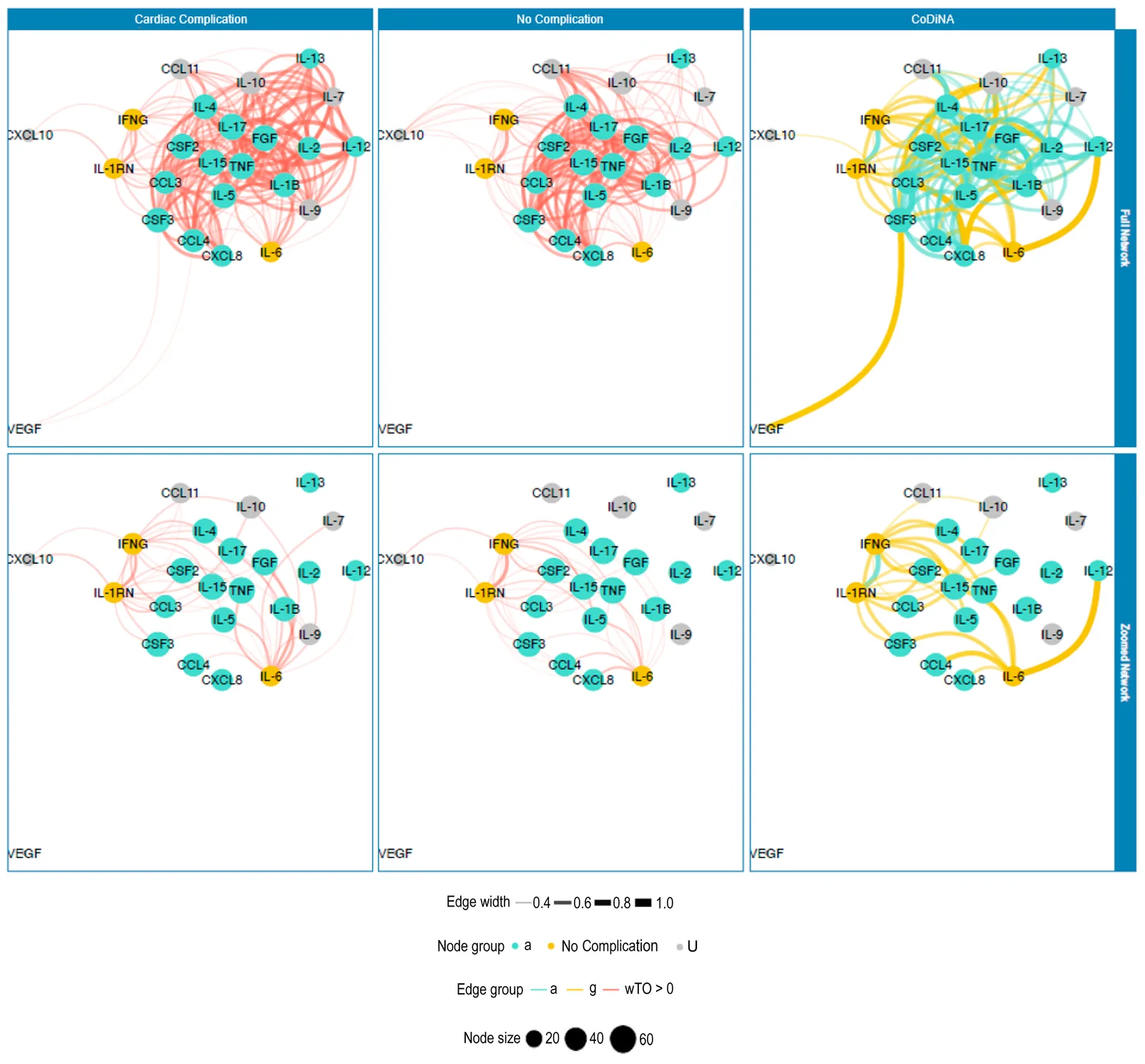
Cytokine network analysis according to cardiac complications. Full (top) and zoomed (bottom) networks are shown for patients with cardiac complications, those without cardiac complications, and the differential CoDiNA network. Patients with cardiac complications exhibited a denser cyto-kine network with a greater number of interactions. CoDiNA identified distinct differential inter-action patterns, particularly involving IFN-γ, IL-1RA, and IL-6. Node colours indicate CoDiNA node groups, node size reflects connectivity, and edge width represents interaction strength. Red edges indicate positive correlations, whereas yellow edges represent differential interactions iden-tified by CoDiNA.

**Table 1:** Clinical characteristics of patients with IE at diagnosis, stratified by in-hospital mortality.

Variables *	Survived n = 119	Died n = 41	*p*-Value
Age (years)	57 [44–66]	63 [52–74]	0.013
Male sex	67 (56)	27 (66)	0.300
**Predisposing condition**			
RHD	23 (19)	9 (22)	0.700
Prosthesis valve	30 (25)	10 (24)	0.900
CIED	43 (36)	14 (34)	0.800
Previous IE	11 (9.2)	7 (17)	0.200
Diabetes mellitus	25 (21)	10 (24)	0.700
Immunosuppression	14 (12)	7 (17)	0.400
Chronic kidney disease	19 (16)	12 (29)	0.063
**Laboratorial finding**			
Hemoglobin (g/dL)	10.60 [9.0–13.1]	10.30 [8.8–12.2]	0.300
Leukocytes (cells/µL)	8525 [6330–14,270]	11,410 [7070–14,180]	0.300
Platelets (number × 10^3^/µL)	224 [160–226]	186 [113–257]	0.150
CRP (mg/L)	56 [22–194]	80 [42–242]	0.061
**Echocardiogram finding**			
Vegetation size (mm)	8 [5–13]	10 [7–15]	0.300
**Overall complications**			
Embolic event at admission	28 (24)	10 (24)	0.900
Embolic events during treatment	20 (17)	14 (34)	0.019
Valvular cardiac surgery	43 (36)	16 (39)	0.700
Surgery indicated but not performed	1 (0.8)	9 (23)	<0.001
Acute kidney injury	22 (24)	22 (65)	<0.001
**Cardiac complications**	42 (35)	31 (76)	<0.001
Valvular regurgitation	38 (32)	20 (49)	0.053
New-onset or worsening HF	47 (40)	32 (78)	<0.001
Cardiogenic shock	15 (16)	21 (62)	<0.001
**Infectious complications**	33 (28)	34 (83)	<0.001
Septic shock	17 (18)	32 (82)	<0.001
Positive cultures after 7 days	6 (6.4)	5 (15)	0.200
Fever persisting for 10 or more days	24 (20)	20 (49)	<0.001

* Data are expressed as the median [interquartile range], or absolute numbers (percentage). Abbreviations: CIED: cardiovascular implantable electronic devices; CRP: C reactive protein; HF: heart failure; IE: infective endocarditis; RHD: rheumatic heart disease.

**Table 2:** Baseline biomarker levels in IE patients according to in-hospital mortality and control group.

Biomarkers	Survived n = 121 *	Died n = 41 *	Control n = 49	*p*-Value
CCL11	11.03 [0.77–32.17]	13.66 [4.12–24.16]	44.69 [21.03–66]	<0.001 ^b,c^
CXCL8	8.64 [4.71–16.88]	18.46 [10.14–59.42]	453.27 [159.06–845.08]	<0.001 ^a,b,c^
CCL3	3.75 [2.02–6.86]	7.66 [3.09–12.06]	13.55 [4.83–83.31]	<0.001 ^a,b,c^
CCL4	22.34 [17.87–29.03]	26.9 [19.3–33.58]	53.89 [34.24–108.08]	<0.001 ^b,c^
CCL2	8.65 [3.75–22.72]	10.45 [5.77–22.64]	23.72 [13.95–59.97]	<0.001 ^b,c^
CCL5	421.49 [269.37–588.61]	345.57 [256.12–513.32]	311.28 [226.46–369.02]	0.006 ^c^
CXCL10	49.74 [22.22–98.88]	55.22 [23.23–151.35]	64.69 [45.76–113.3]	0.101
IL-1 β	0.72 [0.43–1.50]	0.99 [0.54–1.44]	3.17 [1.33–11.13]	<0.001 ^b,c^
IL-6	4.20 [2.25–14]	14.99 [4.22–32.87]	6.12 [1.41–31.84]	0.004 ^a^
TNF-α	17.49 [13.39–23.75]	18.8 [15.26–27.64]	26.54 [18.32–53.63]	<0.001 ^b,c^
IL-12	3.36 [1.39–7.68]	3.36 [2.12–5.40]	5.28 [2.35–7.10]	0.531
IFN-γ	2.28 [0.8–5.99]	3.38 [2.03–8.34]	10.93 [5.53–18.23]	<0.001 ^b,c^
IL-15	165.14 [134.36–190.80]	179.35 [152.59–220.45]	183.23 [145.22–276.82]	0.012 ^a,c^
IL-17	1.98 [0.84–3.65]	2.1 [1.26–5.11]	5.41 [3.11–10.98]	<0.001 ^b,c^
IL-1Ra	173.52 [84.73–371.41]	248.94 [101.59–614.43]	487.84 [242.41–697.04]	<0.001 ^b,c^
IL-4	1.03 [0.41–1.94]	1.18 [0.67–1.99]	2.53 [1.79–3.42]	<0.001 ^b,c^
IL-5	32.49 [20.43–44.84]	40.69 [29.29–61.39]	43.61 [34.36–87.21]	<0.001 ^a,c^
IL-9	22.99 [18.37–27.25]	21.43 [16.18–26.54]	25.86 [20.71–31.53]	0.014 ^b,c^
IL-13	1.35 [0.89–1.92]	1.38 [1.10–2.030]	1.35 [1.16–1.62]	0.515
IL-10	7.3 [4.16–11.52]	8.29 [5.85–13.13]	6.71 [5.34–13.98]	0.358
FGF basic	2.27 [1.53–3.72]	2.75 [1.72–3.89]	4.54 [3.52–9.10]	<0.001 ^b,c^
PDGF	198.76 [69.84–493.72]	164.14 [32.85–350.50]	78.36 [39.75–106.94]	<0.001 ^b,c^
VEGF	34.06 [20.40–75.09]	39.28 [23.26–65.42]	41.7 [29.06–89.68]	0.248
G-CSF	138.14 [79.49–276.97]	302.62 [95.22–535.58]	395.39 [226.32–1763.28]	<0.001 ^a,b,c^
GM-CSF	1.04 [0.78–1.31]	1.15 [0.89–1.40]	1.45 [1.18–2.22]	<0.001 ^b,c^
IL-7	6.82 [4.59–10.08]	6.52 [4.43–10.9]	4.92 [3.36–6.67]	0.002 ^b,c^
IL-2	1.9 [1.25–3.28]	2.61 [1.84–4.83]	3.72 [2.31–11.34]	<0.001 ^a,b,c^

Data are expressed as the median [interquartile range] and correspond to the first sample collected from each patient. This table presents the first sample collected from patients who survived, died, or were from the control group. * Notably, two patients had samples collected during two separate hospital admissions, resulting in 121 samples from 119 patients. The Kruskal–Wallis test was used to assess whether there were statistically significant differences among the three groups. When the *p*-value was <0.05, post hoc pairwise comparisons were performed using the Mann–Whitney U test to identify the groups in which the differences occurred, represented by the letters a, b or c. a = Statistically significant difference between IE patients who died and those who survived. b = Statistically significant difference between IE patients who died and the control group. c = Statistically significant difference between IE patients who survived and the control group. Legend: FGF-basic, basic fibroblast growth factor; G-CSF, granulocyte colony-stimulating factor; GM-CSF, granulocyte-macrophage colony-stimulating factor; IFN-γ, interferon-gamma; IL, interleukin; IL-1Ra, interleukin-1 receptor antagonist; PDGF, platelet-derived growth factor; TNF-α, tumor necrosis factor-alpha; VEGF, vascular endothelial growth factor.

## Data Availability

The datasets presented in this article are not readily available due to privacy and ethical reasons. Requests to access the datasets should be directed to M.C.P.N. at mcarmo@waymail.com.br.

## Supplementary Materials

The following supporting information can be downloaded at https://www.mdpi.com/article/10.3390/tropicalmed11100265/s1: Table S1: Baseline characteristics of infective endocarditis patients; Figure S1: Temporal trajectories of IL-6 levels among patients who experienced at least two of the three complications during hospitalization (embolic, infectious, or cardiac complications).
