# Supplementary material for: Immune Network Dysregulation Predicts Adverse Outcomes in Infective Endocarditis
Source: Trop Med Infect Dis. 2026 Sep 24;11(10):265. doi: 10.3390/tropicalmed11100265 (PMC13709421; doi:10.3390/tropicalmed11100265)
Supplement: Supplementary file 1 [file tropicalmed-11-00265-s001.zip › tropicalmed-4470854-supplementary.pdf]

Supplemental Materials

Table

Table S1. Baseline characteristics of infective endocarditis patients.

Characteristics	
Age (years)	58 [36-80]
Male sex	94 (58.7)
**Predisposing condition**	
CIED	65 (40.8)
Prosthetic valve	40 (25)
Rheumatic heart disease	32 (20)
Mitral valve prolapse	13 (8.2)
Degenerative valve disease	18 (11.3)
Congenital heart disease	10 (6.3)
Previous IE	18 (11.3)
Diabetes *mellitus*	35 (21.9)
Chronic kidney disease	31 (19.4)
Immunosuppressive drug	21 (13.1)
Central venous line	25 (15.6)
Hemodialysis	9 (5.6)
**Clinical presentation**	
Fever	121 (75.6)
Murmur	73 (45.6)
Neurological embolic event	23 (14.4)
Systemic embolic event	25 (15.6)
Peripherical manifestation	13 (8.1)
Splenomegaly	11 (6.9)
**Laboratorial findings**	
Hemoglobin (g/dL)	10.55 [9-12.8]
Leukocytes (cells/μL)	8,690 [6,760-14,267]
Platelets (number x 10^3/μL)	207 [153-262.5]
CRP (mg/L)	67 [26.2-208.3]
Creatinine (mg/dL)	1.1 [0.8-1.62]
**Main causative microorganisms**	
Staphylococcus aureus	37 (23.1)
Coagulase-negative staphylococcus	31 (19.4)
Streptococci	15 (9.4)
Enterococcus sp.	11 (6.9)
Aerobic Gram-negative bacteria	17 (10.6)
Fungi	6 (3.8)
Gram-negative HACEK	1 (0.6)
Negative culture findings	35 (21.9)
**Echocardiographic findings**	
Vegetation size	8 [5.5-14]
Valvular or perivalvular complications*	58 (36.3)
Vegetation location	
- Mitral native	30 (18.8)
- Aortic native	26 (16.6)
- Tricuspid native	11 (6.9)
- Mitral prosthesis	16 (10)
- Aortic prosthesis	11 (8.8)

-	Intravenous line and devices	58 (36.4)

Data are expressed as the median [interquartile range] or absolute numbers (percentage). *Valvular or perivalvular complications include valve leaflet perforation, chordae rupture, prosthetic dysfunction, fistula, and abscess. Abbreviations: CIED: cardiovascular implantable electronic devices; CRP: C reactive protein; HACEK: *Haemophilus*, *Aggregatibacter*, *Cardiobacterium hominis*, *Eikenella corrodens*, and *Kingella kingae;* IE: infective endocarditis.

Figure

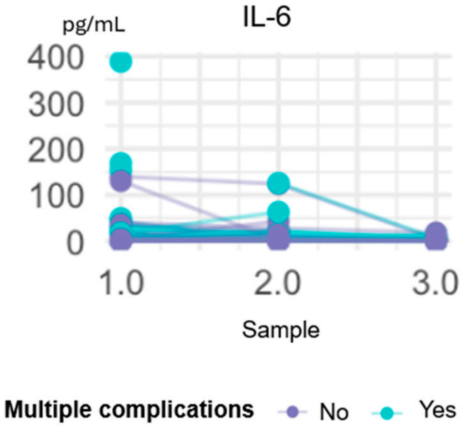

Figure S1. Temporal trajectories of IL-6 levels among patients who experienced at least two of the three complications during hospitalization (embolic, infectious, or cardiac complications).

The numbers on the x-axis correspond to the sampling time points (first, second, and third), while the y-axis represents cytokine levels. Purple indicates IL-6 levels in patients without complications during hospitalization, whereas green indicates levels in patients who experienced at least two of the three complications. IL-6 levels were higher at the first and second sampling time points among patients with multiple complications. Most patients in this group died before the third sample could be collected.
